# Thermal/Optical Methods for Elemental Carbon Quantification in Soils and Urban Dusts: Equivalence of Different Analysis Protocols

**DOI:** 10.1371/journal.pone.0083462

**Published:** 2013-12-17

**Authors:** Yongming Han, Antony Chen, Junji Cao, Kochy Fung, Fai Ho, Beizhan Yan, Changlin Zhan, Suixin Liu, Chong Wei, Zhisheng An

**Affiliations:** 1 Key Laboratory of Aerosol Science & Technology, SKLLQG, Institute of Earth Environment, Chinese Academy of Sciences, Xi’an, China; 2 Desert Research Institute, Reno, Nevada, United States of America; 3 AtmAA Inc., Calabasas, California, United States of America; 4 School of Public Health and Primary Care, The Chinese University of Hong Kong, Shatin, Hong Kong, China; 5 Lamont-Doherty Earth Observatory of Columbia University, Palisades, New York, United States of America; Brandeis University, United States of America

## Abstract

Quantifying elemental carbon (EC) content in geological samples is challenging due to interferences of crustal, salt, and organic material. Thermal/optical analysis, combined with acid pretreatment, represents a feasible approach. However, the consistency of various thermal/optical analysis protocols for this type of samples has never been examined. In this study, urban street dust and soil samples from Baoji, China were pretreated with acids and analyzed with four thermal/optical protocols to investigate how analytical conditions and optical correction affect EC measurement. The EC values measured with reflectance correction (ECR) were found always higher and less sensitive to temperature program than the EC values measured with transmittance correction (ECT). A high-temperature method with extended heating times (STN120) showed the highest ECT/ECR ratio (0.86) while a low-temperature protocol (IMPROVE-550), with heating time adjusted for sample loading, showed the lowest (0.53). STN ECT was higher than IMPROVE ECT, in contrast to results from aerosol samples. A higher peak inert-mode temperature and extended heating times can elevate ECT/ECR ratios for pretreated geological samples by promoting pyrolyzed organic carbon (PyOC) removal over EC under trace levels of oxygen. Considering that PyOC within filter increases ECR while decreases ECT from the actual EC levels, simultaneous ECR and ECT measurements would constrain the range of EC loading and provide information on method performance. Further testing with standard reference materials of common environmental matrices supports the findings. Char and soot fractions of EC can be further separated using the IMPROVE protocol. The char/soot ratio was lower in street dusts (2.2 on average) than in soils (5.2 on average), most likely reflecting motor vehicle emissions. The soot concentrations agreed with EC from CTO-375, a pure thermal method.

## Introduction

Elemental carbon (EC, often referred to as black carbon, BC, in soil and sediment research) is produced from incomplete combustion of biomass or fossil fuel [1,2,3]. EC is not a well-defined material; rather it comprises a spectrum of carbonaceous materials that can be viewed as a “continuum” from char, i.e., partially-combusted solid residues, to highly graphitized soot − clusters of carbon particles formed via gas-phase processes [1,2,4]. EC plays an important role in the global carbon cycle [2], the Earth’s radiative balance [5], and human health [6]. In addition, biochar, an engineered BC from pyrolysis of biomass that is often used as pre-dry biomass feedstock and charcoal briquettes, contributes to environmental benefits such as mitigation of climate change, improvement of soils, and reduction of environmental pollution in both natural and agricultural ecosystems [7,8]. 

There is still no universally accepted method for EC quantification. Comparisons of different methods for measuring EC have been conducted in the last decade for geological materials [9,10,11] and for aerosol samples [12,13,14,15]. Different methods were shown to report a wide range of EC concentration (e.g. differences of up to 571 times for soils and sediments [11] and up to a factor of 7 for a given aerosol samples [12]). This has been attributed to two factors: 1) the incorrect identification of non-EC as EC and vice versa and 2) large variations in selectivity of the various techniques across the EC continuum [10]. For both geological and aerosol samples, matrix effects contribute to the inconsistencies among methods; indeed some methods have shown higher EC for one set of samples but lower EC for the others relative to a common benchmark [12].

Thermal/optical methods are the most widely used and accepted approach for aerosol EC analysis [12,16]. A variety of modifications to these methods such as the IMPROVE (Interagency Monitoring of Protected Visual Environments) [17,18], NIOSH (National Institute of Occupational Safety and Health) [19], STN (Speciation Trends Network, a modification of NIOSH) [20] and EUSAAR (European Supersites for Atmospheric Aerosol Research) [21] protocols, have been developed in the last three decades. The methods are based on that low-volatility EC is not liberated in an inert atmosphere under temperatures >350°C; this allows the more volatile organic carbon (OC) to be separated from EC. Typically two phases of heating are implemented on aerosol particles collected on filters. First, OC evolves in inert atmosphere, where pyrolysis may occur. Since pyrolyzed organic carbon (PyOC) is artificial EC created in the measurement process, a laser is used to monitor the PyOC formation through the decrease of filter reflectance or transmittance to perform an “optical correction”. The second phase involves heating in an oxidizing atmosphere in which both EC and PyOC are combusted. An organic pyrolysis (OP) fraction is defined as the carbon that evolves after the introduction of oxygen and before the laser signal (reflectance or transmittance) returns to its initial value (i.e., the crossover or split point). EC is quantified as the carbon evolved from the second phase minus OP.

PyOC formation is found to be sensitive to heating temperature and duration in the inert phase which vary among different analysis protocols [22]. This issue along with PyOC and EC’s attenuation of laser light with different efficiencies [14,22] causes the diversity in EC quantification. Moreover, salts such as chloride that mix with soot particles are known to cause evolution of soot at relatively low temperatures [23], while carbonate and metal oxides can evolve when temperatures are higher than 400°C [24], releasing oxygen to oxidize OC, PyOC, and EC in inert atmosphere. This oxidation sometimes leads to “early split,” that is, when crossover occurs before the introduction of oxygen and zero or negative OP is reported. Therefore, the operationally-defined OP fraction is neither indicative of nor necessarily correlated with the actual PyOC amount. 

The commonly used method for quantifying EC in soils and sediments is the chemo-thermal oxidation (e.g., CTO-375) method. EC measured using CTO-375 is often a low estimate [9,10], representing only the soot fraction of EC [25], as char material is entirely or partially excluded [26]. In recent year, thermal/optical methods were introduced for measuring EC in sediments and soils in conjunction with acid pretreatment to minimize interferences from carbonate, metal oxides, salts, and water soluble organic compounds (WSOC) [18,27]. This allows better comparability between EC contents measured in geological material and aerosols [27]. These data have been used to reconstruct the EC pollution history in Eastern China [9] and infer historical trends in atmospheric EC at Whiteface Mountain, New York, USA [27]. 

How EC quantification depends on thermal/optical analysis protocol has not been evaluated systematically for geological material. Knowledge learned from aerosol studies may not be extrapolated to these samples. In this study, surface soil samples with relatively low expected EC contents and street dust samples with relatively high expected EC contents (from motor vehicle exhausts and coal combustion) were collected in the central China. These samples as well as three standard reference materials (SRMs) were used to compare EC concentrations determined using a pretreatment procedure coupled with IMPROVE, STN, and CTO-375 protocols. These protocols were further modified to evaluate the influence of analytical parameters as well as EC measurement uncertainty. 

## Methodology

### 2.1: Sample collection and pretreatment

Twelve street dust samples from paved roads and 13 surface soil samples from the top 5 cm of unpaved surfaces were collected in Baoji city, Shaanxi Province, China (see Figure S1), using a clean polypropylene dustpan along with a brush or a shovel. The samples were collected in public areas with no specific permission required and the field studies did not involve endangered or protected species. All samples were dried in an oven at 40°C for 2 days. The dried samples were ground and homogenized with an agate mortar and sieved though a 200 mesh sieve (66 µm). Three SRMs of environmental matrices: including urban dust from Washington D.C (SRM-1649a) and clay rich soils (Mollisol and Vertisol) [10,18] were also prepared as controls of the experiment. 

Small portions of samples (60-110, 200-300 and 8-40 mg for each urban dust, soil, and SRM samples, respectively) were used for the analyses. The sample pretreatment procedure followed that of Han et al. (2007b; 2009b): hydrochloric (HCl) and hydrofluoric (HF) acids were used to remove carbonate, metal oxides, and silicates while deionized water (electrical resistivity of 18.2 Ω and TOC < 5 mg L^-1^) was used to wash off the ions, WSOC and acids from the residues. The sample residues were then filtered through pre-fired (850°C for 3 hours) quartz-fiber filters (0.4 µm pore size, Whatman) and air dried in an oven (35°C for 8 hours). The homogeneity of sample residues on filters has been verified in Han et al. (2007b). Each filter was cut into four quarters for EC analysis with different methods (3/4 for IMPROVE and STN, and 1/4 for CTO).

### 2.2: Thermal analysis protocols

**Figure 1:**
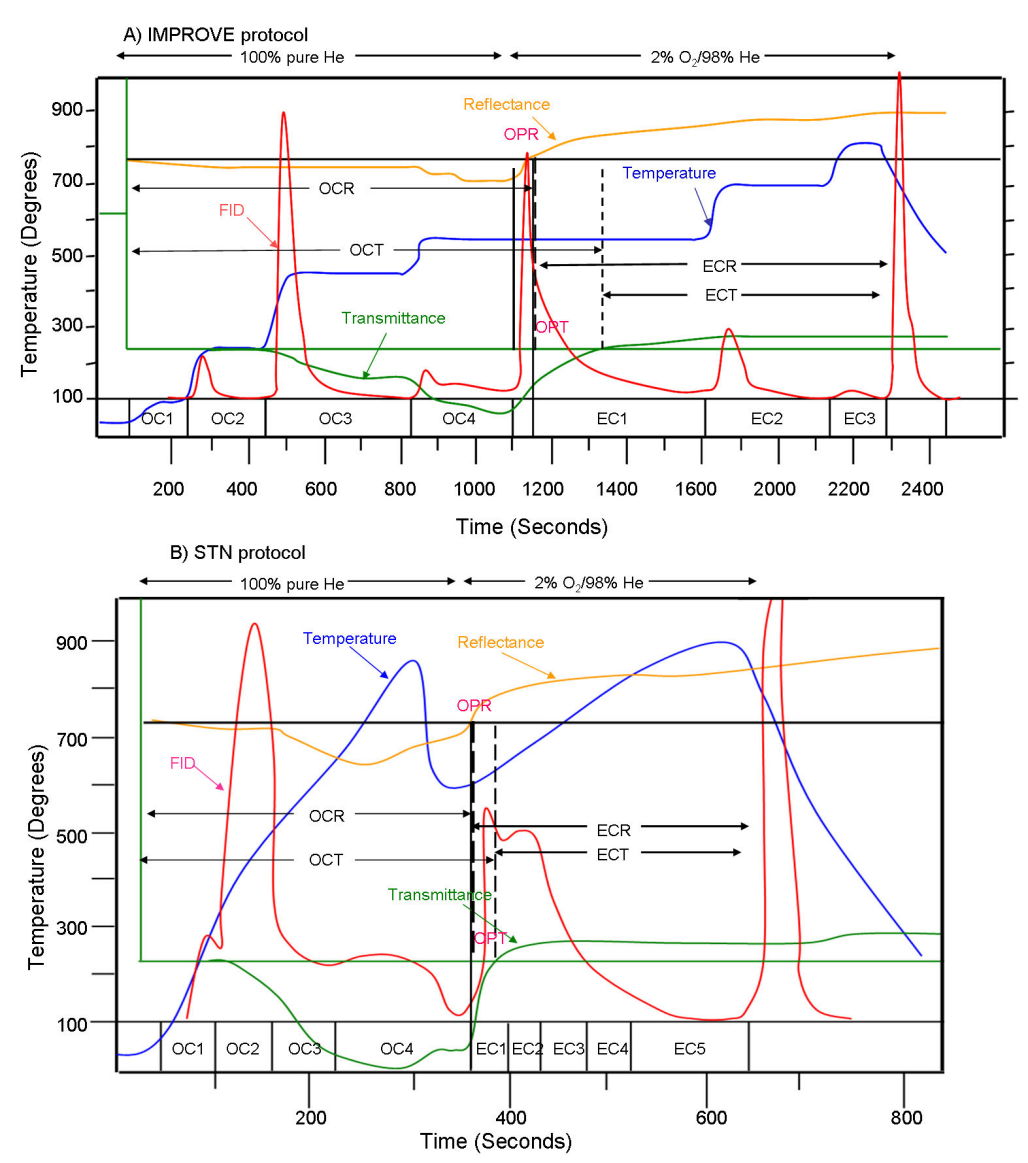
Thermograms of A) the conventional IMPROVE (Interagency Monitoring of Protected Visual Environments) and B) the STN (Speciation Trends Network) protocols (for sample UD-2).

As detailed in Table S1, the IMPROVE protocol reports four OC fractions (OC1 to OC4 at 120, 250, 450 and 550°C in a pure helium atmosphere), three EC fractions (EC1 to EC3 at 550, 700 and 800°C in 2% oxygen/98% helium atmosphere), and one OP fraction while the STN protocol also reports four OC and one OP fractions, as well as five EC fractions. The peak inert-mode temperature of STN (900°C) is much higher than that of IMPROVE (550°C). The residence time at each temperature step for the IMPROVE analysis varies between 150 and 600 s depending on sample loading, while for STN the residence times are pre-specified (45–120 s) and relatively short. IMPROVE also differs from STN in optical correction as IMPROVE uses laser reflected from the filter (R) instead of transmitted through the filter (T) to infer the PyOC formation and oxidation (see Figure 1[A] and [B] for descriptions and typical thermograms of the IMPROVE and STN protocols).

The IMPROVE and STN protocols were further modified in this study to investigate the influence of temperature and residence time on EC determination. For IMPROVE the temperatures of OC4 and EC1 were increased from the normal 550°C (IMPROVE-550) to 675°C (IMPROVE-675), and for STN the time intervals of 60 seconds for OC1 to OC4 (STN60) were increased to 120 s (STN120). In the EUSAAR protocol, which seeks to reconcile the IMPROVE and STN protocols, the last OC step heats samples in an inert atmosphere up to 650°C for 180 s (Table S1). 

The CTO-375 method preheats samples in a muffle furnace at 375°C for 18 h and then quantifies the remaining carbon in the residues as EC [28,29]. A DRI Model 2001 Thermal/Optical Carbon Analyzer (Atmoslytic Inc. Calabasas, CA) was used to implement all the (IMPROVE, STN and CTO-375) protocols. This instrument can monitor both R and T of a filter throughout the analysis [30], yielding reflectance-corrected EC (ECR) and transmittance-corrected EC (ECT) for IMPROVE and STN protocols (see Figure 1, though IMPROVE ECR and STN ECT are those conventionally reported). For CTO-375, the residual carbon after preheating was determined using the IMPROVE protocol, and it was defined as CTO-375 EC [9,31]. 

## Results and Discussion

Results from the analysis of 12 urban dust and 13 soil samples after acid pretreatment using the IMPROVE and STN protocols, as well as their modifications are presented in Table S2. The urban dust samples from Baoji show 10 to 100 times higher EC concentrations than the soil samples. This is consistent with a previous study conducted in Xi’an (a major Chinese city ~155 km east of Baoji) where the average EC concentration in urban dust was 7.2 mg g^-1^, ~10 times what was measured in soil [32]. The total carbon (TC, i. e. OC + EC) concentrations measured with all the conventional and modified protocols on different sample punches were similar (within ±5%, see Figure S2 and Table 1), confirming the homogeneity of residuals on the filters. To minimize the influence of outliers, a robust regression algorithm [33] that applies iteratively re-weighted least squares approach was achieved with the Matlab® “robustfit” function and used in this and all other analyses throughout the study.

**Table 1:** Statistical comparisons among carbon fractions measured with the thermal/optical reflectance (TOR) and transmittance (TOT) protocols and the CTO-375 method (see Table S1 for experimental parameters).

**Protocols**		**Robust linear regression ^*a*^**				**Ordinary linear regression ^*a*^**			**Number of pairs**	**Average ratio of y/x±SD**	**Average (mg g^-1^)**			**kruskalwallis-test**
**x**	**y**	**Intercept**	**Regression**	**Number of outliers**	**R^2^**	**Intercept**	**Regression**	**R^2^**						
		**(mg g^-1^)**	**slope**			**(mg g^-1^)**	**slope**				**x**	**y**	**y-x**	**p-value ^*b*^**
**ECR**	**ECR**													
**IMPROVE550**	**IMPROVE675**	0.13	1.01	4	1.00	0	0.99	1.00	25	1.08±0.13	7.94	8.03	0.09	0.69
**IMPROVE550**	**SIN60**	0.08	1.03	7	1.00	0	1.06	1.00	25	1.09±0.09	7.94	8.51	0.57	0.61
**IMPROVE550**	**SIN120**	0.18	0.96	5	1.00	0	1.01	1.00	25	1.09±0.09	7.94	8.26	0.32	0.55
**ECT**	**ECT**													
**IMPROVE550**	**IMPROVE675**	-0.26	1.27	2	0.99	0	1.28	0.98	25	1.16±0.20	4.53	5.68	1.15	0.63
**IMPROVE550**	**SIN60**	-0.2	1.47	5	1.00	0	1.44	0.98	25	1.30±0.23	4.53	6.43	1.9	0.31
**IMPROVE550**	**SIN120**	-0.24	1.64	2	0.99	0	1.62	0.98	25	1.42±0.30	4.53	7.19	2.66	0.19
**TC**	**TC**													
**IMPROVE550**	**IMPROVE675**	0.05	1.01	3	1.00	0	1.01	1.00	25	1.01±0.03	14.76	14.89	0.13	0.85
**IMPROVE550**	**SIN60**	-0.03	1.02	4	1.00	0	1.00	1.00	25	0.99±0.06	14.76	14.64	-0.12	0.99
**IMPROVE550**	**SIN120**	0.05	0.99	1	1.00	0	1.00	1.00	25	1.00±0.06	14.76	14.72	-0.04	0.99
**ECR**	**ECT**													
**IMPROVE550**	**IMPROVE550**	0.28	0.51	2	1.00	0	0.53	0.98	25	0.69±0.14	7.94	4.53	-3.41	0.15
**IMPROVE675**	**IMPROVE675**	-0.02	0.72	1	1.00	0	0.7	1.00	25	0.73±0.13	8.03	5.68	-2.35	0.26
**SIN60**	**SIN60**	0.12	0.74	3	1.00	0	0.73	1.00	25	0.80±0.08	8.51	6.43	-2.08	0.38
**SIN120**	**SIN120**	0	0.86	6	1.00	0	0.86	1.00	25	0.87±0.04	8.26	7.20	-1.06	0.49
**ECR/ECT/soot**	**EC**													
**IMPROVE-550 ECR**	**CTO-375**	0.08	0.04	6	0.96	0.30	0.05	0.44	25	0.13±0.08	7.94	0.71	-7.23	<0.01
**IMPROVE-550 ECT**	**CTO-375**	0.06	0.08	6	0.97	0.25	0.1	0.44	25	0.19±0.12	4.53	0.71	-3.82	<0.01
**STN60 ECR**	**CTO-375**	0.1	0.04	7	0.95	0.30	0.05	0.41	25	0.12±0.08	8.51	0.71	-7.8	<0.01
**STN60 ECT**	**CTO-375**	0.07	0.06	8	0.98	0.28	0.07	0.41	25	0.15±0.09	6.43	0.71	-5.72	<0.01
**IMPROVE-550 soot**	**CTO-375**	0.09	0.3	8	0.99	0.37	0.24	0.34	25	0.88±0.57	1.4	0.71	-0.69	0.48

^a^ Robust linear regression weights variable by precisions in both independent and dependent variables. Ordinary linear regression does not weight variables by their precisions;

^b^ Probability of whether protocol x and protocol y yield similar or different results. For example, a probability of 0.99 would mean there is a 99% likelihood that protocol x results are similar to protocol y results. A probability of 0.01 or less would mean that protocol x results are most likely different from protocol y results.

### 3.1: Comparison of optical pyrolysis corrections on EC determinations

ECR was found higher than ECT regardless of sample loading and analysis protocol (Figure S3 and Table 1). Transmittance returned to the baseline later than reflectance due to some of PyOC within the filter that evolved later than EC on the filter surface, as described in previous aerosol studies [22,34,35]. The ECR and ECT concentrations, however, correlated well with each other with R > 0.97, implying only multiplicative biases between the reflectance and transmittance pyrolysis corrections. The correlation coefficients are comparable to or better than those from aerosol studies [22,34]. Note the pretreated dusts/soils represented a simpler matrix than aerosol samples as the pretreatment removed metal oxides [36], salts [23] and carbonates [24], minimizing the interferences to EC measurement. 

The IMPROVE-550 protocol produced the lowest regression slope (0.53) between ECT and ECR (Table 1). This value is lower than the IMPROVE-550 ECT/ECR slope of 0.67 for ambient aerosol samples reported by Cheng et al. [37]. Under the IMPROVE-550 protocol, urban street dusts (with relatively high soot levels, Table S2) yielded a lower average ECT/ECR ratio than soil samples (with low soot contents) (see Figure S4). This suggests that chemical composition of a sample, such as the relative abundance of OC and EC, can influence EC quantification through its effect on analysis time duration and/or pyrolysis. The STN120 protocol reported the highest ECT/ECR ratio with little difference between the urban dust and soil samples (i.e., ECT/ECR slope of 0.88 and 0.85, respectively). 

### 3.2: Comparison of inter-protocol ECR and ECT concentrations

Previous aerosol studies [22,35] showed that STN (STN60 in this paper) ECR concentrations agree well (within ±10%) with their corresponding IMPROVE (IMPROVE-550 in this paper) ECR values; but STN ECT is often lower than the IMPROVE ECT. The STN protocol generates more PyOC than IMPROVE, including that within the filter, due to more rapid heating steps in the inert atmosphere. As reflectance is not sensitive to PyOC within the filter, some of which does not evolve until the crossover point would be classified as EC (a positive artifact for ECR). This is likely a minor fraction of carbon relative to EC for most aerosol samples as evidenced by similar IMPROVE and STN ECR (e.g., with the difference less than the replicate precision of IMPROVE or STN ECR). 

Transmittance attenuation caused by PyOC is not negligible at the reflectance crossover, and so the transmittance crossover point is delayed (Figure 1). Transmittance is sensitive to both EC and PyOC on the filter surface and PyOC within the filter; the latter is known to have a much higher mass-specific absorption efficiency than EC (a negative artifact for ECT) [14,22,34]. The lower STN60 ECT compared with the IMPROVE-550 ECT would be consistent with more within-filter PyOC left at the reflectance crossover and less surface EC left at the transmittance crossover point.

ECR (or ECT) of pretreated soil/dust samples, as determined by the IMPROVE-550 and STN60 protocols, are compared for the first time in this study. To test whether the inter-protocol differences are caused by the heating temperature and/or rate, ECR and ECT from IMPROVE-675 and STN120 are also presented and compared. 

#### 3.2.1: Effects of maximum inert-atmosphere temperature

**Figure 2:**
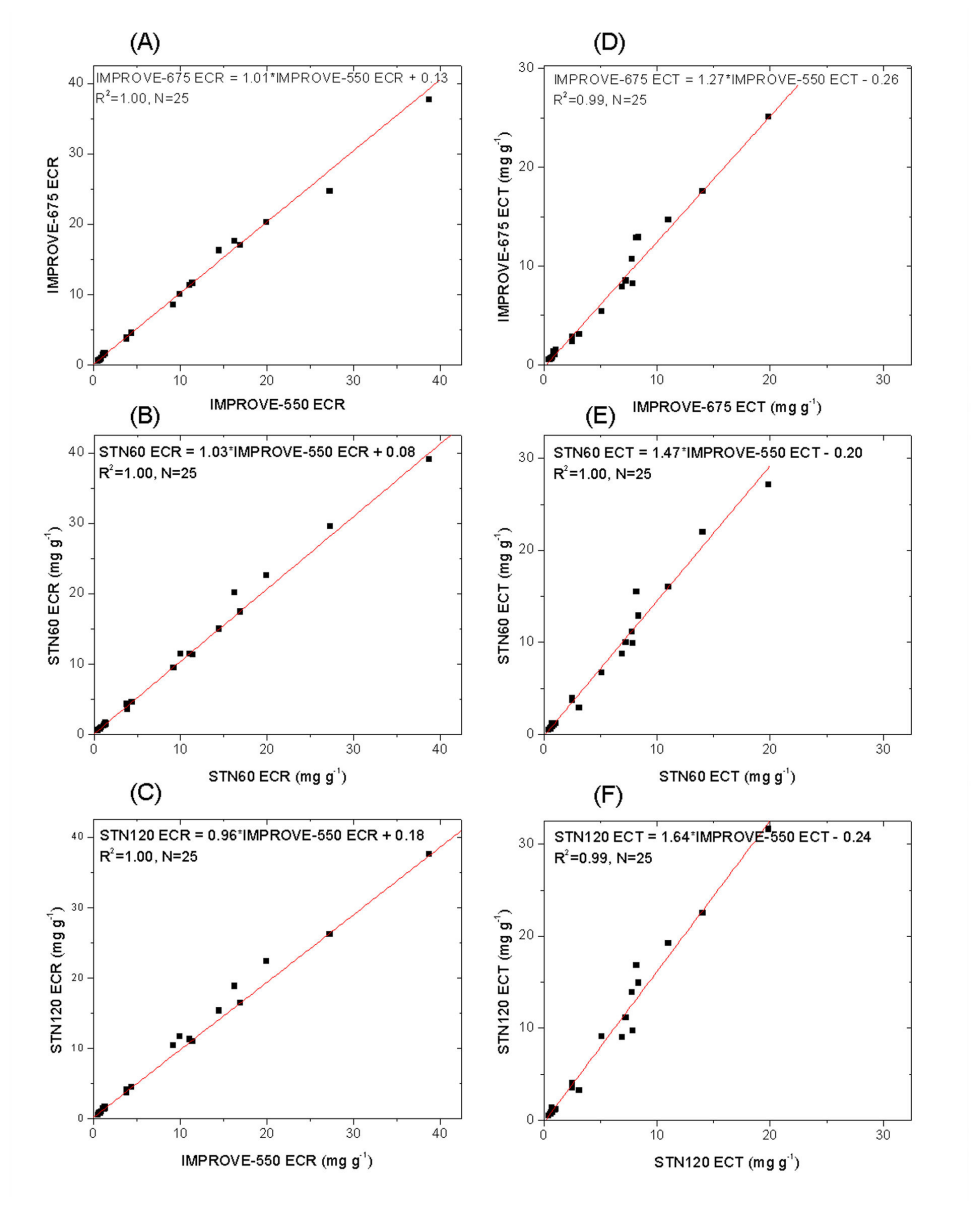
Comparison of ECR and ECT among different protocols. (A)-(C): Comparison of ECR (EC with reflectance correction) between IMPROVE-550 and other three protocols, which shows similar ECR concentrations among different protocols; (D)-(F): Comparison of ECT (EC with transmittance correction) between IMPROVE-550 and other three protocols, which indicates that with the increase in peak inert-mode temperature and analysis time duration, ECT concentrations increase. Robust linear regression was used for all the analyses.

**Figure 3:**
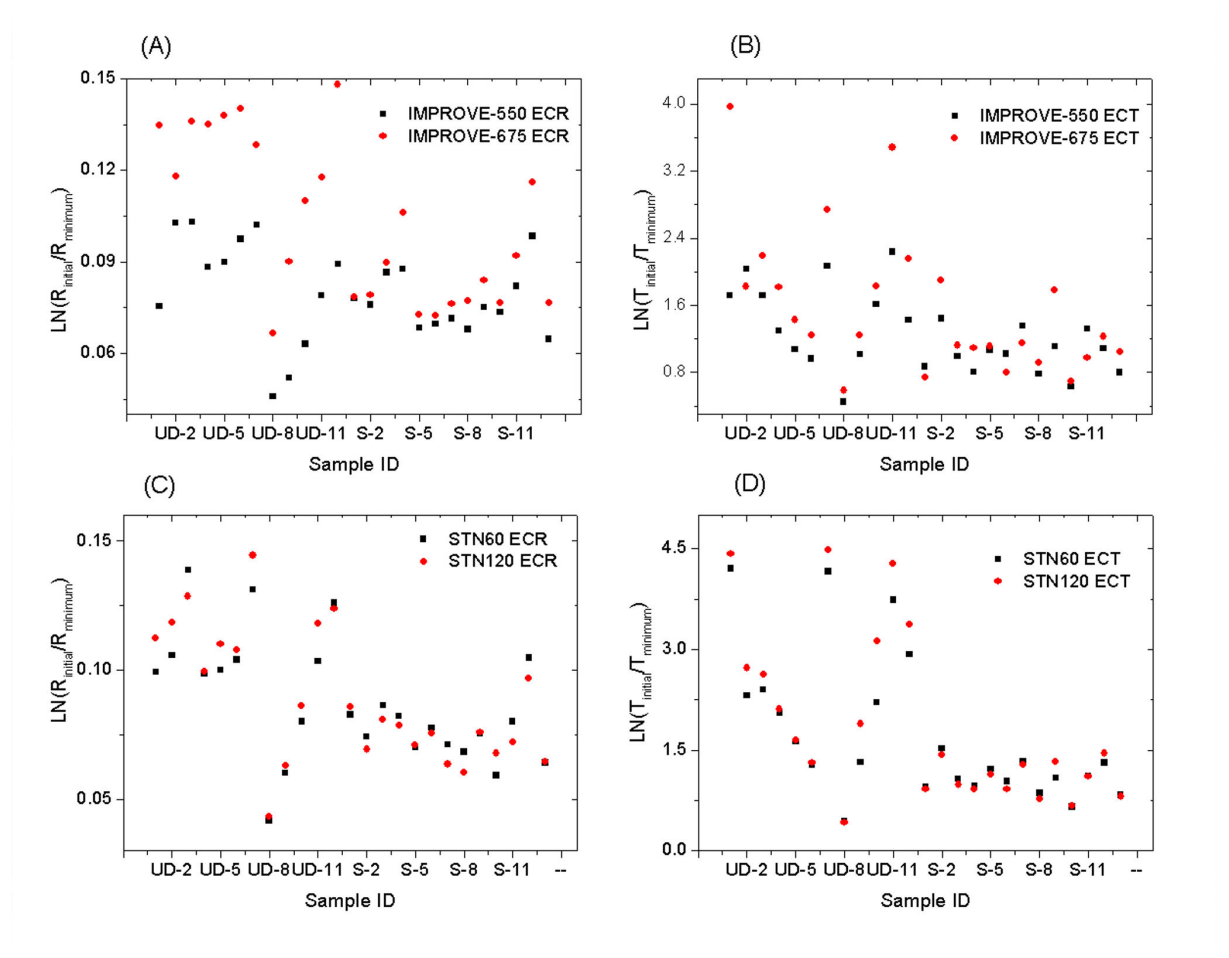
Laser signal changes, i.e., ∆R_min_ and ∆T_min_ (the minimum R and T minus the corresponding initial R and T) among the four different protocols (IMPROVE-550 vs. IMPROVE-675 in (A)-(B) and STN60 vs. STN120 in (C)-(D)), which serve as indicators for the degree of pyrolysis. UD stands for urban dust and S stands for soil. See supplement Figure S1 and Table S1 for sample IDs.

A comparison of IMPROVE-550 and IMPROVE-675 results suggests no significant changes in ECR (p > 0.05) when increasing the highest temperature in the inert atmosphere from 550°C to 675°C, although ECT increases by > 20% on average (p < 0.01) (Figure 2 and Table 1). This is consistent with less pyrolysis at the R crossover for IMPROVE-675 leading to a lower (negative) artifact for ECT. However, more PyOC production with the IMPROVE-675 protocol is evident according to the minimum R and T relative to their initial values, i.e. ∆R_min_ and ∆T_min_ (the minimum R and T values minus their corresponding initial R and T, indicators for the PyOC level). Both ∆R_min_ and ∆T_min_ are generally lower (i.e., darker) for IMPROVE-675 compared with IMPROVE-550 (see Figure 3).

The seemingly contradictory findings point to a rapid removal of PyOC, especially those within the filter, during the inert 675°C heating step. Cheng et al. (2012a) hypothesized two competing effects when the peak inert-atmosphere temperature was raised in thermal analysis – one tended to increase the amount of PyOC while the other caused more PyOC to evolve before EC. That hypothesis was supported by rapid decreases in R and T during the heating from OC3 to OC4 followed by gradual increases of R and T throughout the OC4 step, as was often observed in the STN analysis with a high OC4 temperature (e.g., Figure 1B). 

The first of the competing effects would increase ECR slightly and lowers ECT substantially as described by Chow et al. (2004). The second effect, contrarily, would lower ECR and increase ECT, and in extreme cases bring ECR and ECT into agreement (i.e., ECR ~ ECT). The relative strengths of the two effects may depend on the sample matrix and time duration of the OC4 step. For the dust and soil samples in this study, the second effect appears to be more important because the IMPROVE-675 ECT concentrations were higher than those from IMPROVE-550. Results like this had only been observed for solvent-extracted aerosol samples [38], but the acid-pretreated samples in this study may share similar characteristics.

Although the mechanism(s) responsible for the removal of PyOC in the inert atmosphere is unclear, it would require trace levels of oxygen in the analyzer oven. Since the minerals in our dust and soil samples have been removed nearly completely, the trace amount of oxygen probably resulted from the penetration of ambient air, which could cause up to 100 ppmv of oxygen [39]. In the 2% oxygen/98% helium atmosphere (during the second phase of heating), EC evolves faster than PyOC within the filter [22,34], but the relative oxidation rates of PyOC and EC in <100 ppmv oxygen environment may be very different.

**Figure 4:**
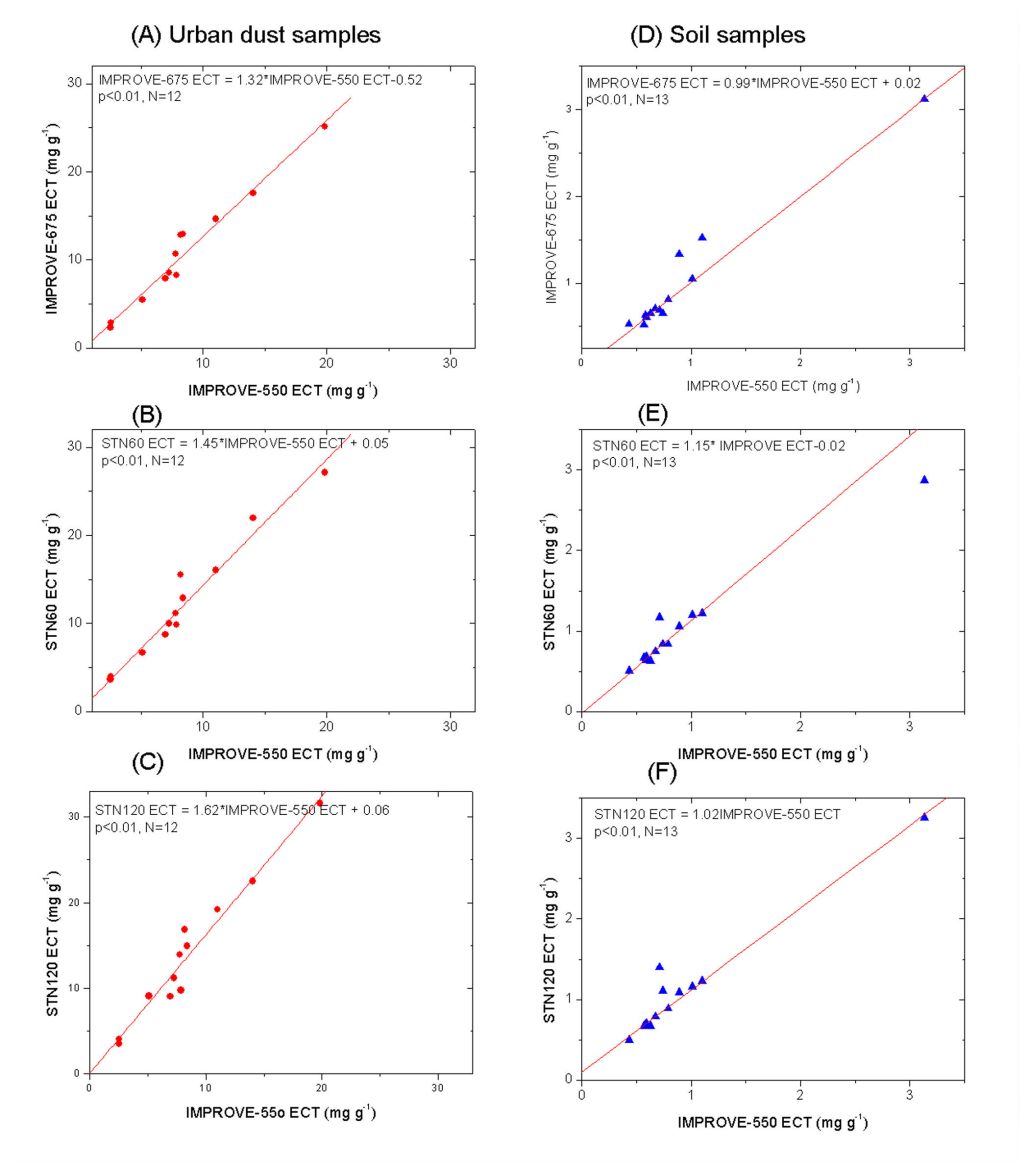
Comparison of ECT (EC with transmittance correction) between IMPROVE-550 and other three protocols using robust linear regression. (A)-(C): in urban dust samples with relatively high percentage of soot contents and (D)-(F) in soil samples with relatively low soot contents.

It should be noted in Figure 4 that the relatively higher IMPROVE-675 ECT is driven by the urban dust samples. For soil samples alone, the changes in ECR and ECT are not significant between the IMPROVE-550 and IMPROVE-675 protocols. This is consistent with minor changes in the amount of pyrolysis between the two protocols as indicated by the initial and minimum laser signals (i.e., samples S-1 to S-13 in Figure 3). 

#### 3.2.2: Effects of inert-atmosphere heating duration

Results of STN60 and STN120 analyses suggest that the increased time duration in the inert atmosphere (from 60 to 120 seconds) had little if any effect on ECR, but did increase the ECT concentrations by an average of 12% (p < 0.01). The changes in ∆R_min_ and ∆T_min_ were not significant between the STN60 and STN120 protocols (Figure 3). Although the PyOC production does not increase with the duration of heating, STN120 has slightly less PyOC left at the R crossover and more EC left on the filter surface at the T crossover point (i.e., smaller artifact and higher ECT) compared with STN60.

 The higher inert-atmosphere temperatures (up to 900°C) used in the STN procedure promotes oxidation of PyOC and EC in the inert atmosphere with trace levels of oxygen. Thus the second aforementioned effect, i.e., preferential evolution of PyOC to EC, may be strengthened further by increasing the time duration from 60 to 120 seconds. This explains why less PyOC is left within filter at the R crossover point and subsequently higher ECT as ECT is dominated by surface EC with a lower mass-specific absorption efficiency. In fact, the R crossover point for STN120 often occurred before the introduction of oxygen. The differences in ECT between STN60 and STN120 were smaller for soil samples than street dusts (Figure 4), consistent with less pyrolysis in the soil samples.

### 3.3: Implications for analysis protocol performance

According to ∆R_min_ and ∆T_min_ values (Table S3), changes in R and T are smaller during the analysis with IMPROVE-550 than with STN60. Clearly the conventional STN protocol generates more PyOC than IMPROVE. At their respective R crossover points, there should be more PyOC left within the filters analyzed by STN60 than those by IMPROVE-550. STN60 ECR is indeed slightly higher than IMPROVE-550 ECR (Figure 2 and Table 1). At their respective T crossover points, however, STN60 ECT appears to contain less within-filter PyOC and higher surface EC than IMPROVE-550 ECT; as a result, STN60 ECT > IMPROVE-550 ECT (Figure 2). The observation also supports a more rapid evolution of pyrolyzed carbon between the R and T crossovers during the STN60 analysis compared with the IMPROVE-550 analysis. 

Concerns over the IMPROVE thermal optical/reflectance (TOR) protocol include 1) the temperature is too low to evolve all of the OC in the inert atmosphere and 2) some of the within-filter PyOC is not monitored by reflectance. Either of these problems results in artificially high ECR concentrations. The IMPROVE_A protocol [39] that increases the OC4 temperature to 580°C alleviates the first problem to some degrees. The main concern associated with the STN thermal-optical transmittance (TOT) protocol, on the other hand, is that the higher absorption efficiency of PyOC relative to EC would tend to decrease the ECT concentration. Since both ECR and ECT are influenced by the amount of PyOC, future protocol development should consider minimizing PyOC formation and/or removing PyOC faster than EC after it is formed. With the increase of temperature and heating duration in the inert atmosphere, the ECR and ECT concentrations become much more similar for the acid-pretreated samples (with ratios increasing from 0.53 to 0.86). For other (aerosol) samples, however, such conditions could lead to the opposite result [22]. Nonetheless, ECR and ECT provide the upper and lower bounds, respectively, of actual EC concentration if the thermal optical method can measure the real EC concentrations.

The EUSAAR protocol [21] seeks to mitigate the problem inherent to STN by lowering the highest temperature in He to 650°C and extending the analysis time. The lower temperature reduces pyrolysis while the longer time duration in the inert atmosphere promotes PyOC removal prior to EC. Among the four temperature protocols investigated in this study, IMPROVE-675 is most similar to EUSAAR, but yet it is the STN120 produces the closest ECR and ECT results and smallest bounds for actual EC concentration. This implies that the maximum inert-atmosphere temperature for the IMPROVE and EUSAAR procedures might need be raised further for analysis of the pretreated dust and soil samples.

### 3.4: Comparability of the thermal/optical and CTO methods

**Figure 5:**
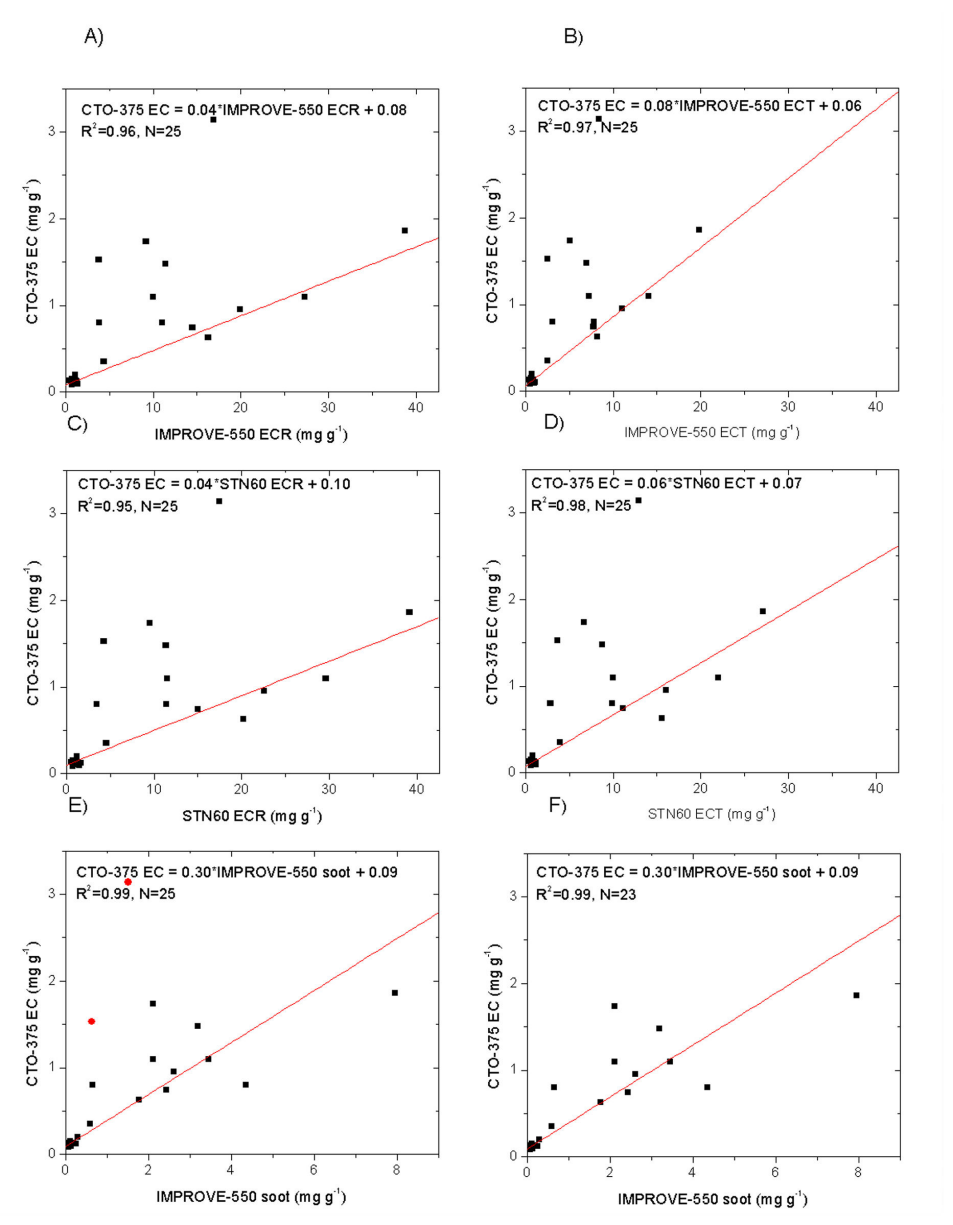
Comparison of CTO-375 EC with A) IMPROVE-550 ECR, B) IMPROVE-550 ECT, C) STN60 ECR, and D) STN60 ECT. The relationship between CTO-375 EC and IMPROVE soot (EC2+EC3 by IMPROVE-550) for E) all dust and soil samples as well as F) those excluding two samples (red circles in E) with high EC1 are also presented. All concentrations are in mg g^-1^. Robust linear regression was used for all the analyses.

A comparison of CTO-375 EC with the IMPROVE-550 and STN60 ECR/ECT shows only moderate correlations (R = 0.64 - 0.68), and CTO-375 EC is generally biased low relative to those from the thermal/optical methods (Figure 5 and Table 1 for ordinary regression). This is consistent with CTO-375 being specifically designed to measure soot [28,40], which only accounts for a small fraction of EC in our samples, although charring sometimes occurs [26]. 

Han et al.[31] showed that the IMPROVE protocol could be used to differentiate char (operationally defined as the low-temperature EC fraction, EC1 - PyOC, see Figure 1A) from soot (defined as the high-temperature EC fractions, EC2 + EC3). Char and soot show markedly different concentrations between urban dust and surface soil samples, with urban dusts showing much higher (more than 10 times on average) concentrations than surface soils, similar to the results from Xi’an [32]. The char/soot ratio in urban dusts averaged at 2.24, significantly lower (p < 0.01) than the ratio found for surface soil samples (averaging at 5.23). This can be explained by a stronger influence of motor vehicle exhausts on the urban street dusts as fossil fuel combustion typically produces a lower char/soot ratio than biomass burning [41].

The IMPROVE-550 soot and CTO-375 EC of surface soil samples are comparable, with the ratios of IMPROVE-550 soot to CTO-375 EC ranging from 0.7 to 1.5 (with one exception of 2.1). For urban dust samples, however, the CTO-375 EC values were generally much lower than the corresponding IMPROVE-550 soot concentrations. CTO-375 applies low temperature but long-term heating (18 h) to separate EC from the other carbon components, and some of the soot can be oxidized/removed in the process [26]. CTO-375 likely underestimated soot in the urban dust samples. There are two dust samples (Numbers UD-8 and UD-11), showing higher CTO-375 EC than IMPROVE-550 soot. EC1 fractions were relatively high in those two samples, and the CTO-375 method may not remove the char completely [10]. Excluding the two samples with high EC1, the correlation coefficient of CTO-375 EC with IMPROVE-550 soot would improve significantly to 0.84 (p<0.01, Table 1 for ordinary regression).

### 3.5: Different protocol performances on SRMs

 The EC concentrations of SRMs measured by different protocols were presented in Table 2, which are comparable with previous studies [10,18]. ECR concentrations are again very similar among different protocols. As for ECT, the urban dust SRM-1649a confirms that the increase in peak inert-mode temperature and analysis time duration increases EC concentrations (Table 2), while the two soil samples with low soot contents display minor variations in ECR and ECT concentrations, consistent with the findings from Baoji soil samples (Figures 4 and S4). 

**Table 2:** EC concentrations (in mg g ^-1^) for environmental standard reference materials measured with different thermal/optical protocols.

Environmental matrix	Reference ID	IMPROVE-550			IMPROVE-675		STN60		STN120		CTO-375
		ECR	ECT	Soot	ECR	ECT	ECR	ECT	ECR	ECT	EC
Urban dust from Washington D.C.	SRM-1949a	49.69±1.21	31.34±1.18	5.63±0.12	48.58±2.11	35.63±1.31	50.04±2.35	41.46±1.98	50.71±2.18	45.23±1.76	7.04±0.73
Wiesenboden Australia soil	Vertisol	13.80±1.42	13.29±1.42	0.12±0.01	13.03±1.11	11.49±1.23	12.69±0.87	12.62±1.10	12.93±1.03	12.86±0.98	0.30±0.03
Chernozerm Germany soil	Mollisol	6.70±0.58	6.39±0.52	0.08±0.01	5.90±0.46	4.80±1.42	6.00±0.62	5.44±0.39	6.20±0.50	5.72±0.32	0.17±0.02

The values are reported as average ± standard deviation from triplicate measurements.

## Implications and Conclusion

Applying thermal/optical methods to the analysis of aerosol and soil/sediment samples bridges atmospheric and geological EC measurements and allows reconciliation of short-term and long-term EC records. The acid pretreatment of geological samples removes most of the interfering materials, and this makes the samples suitable for EC analysis with thermal/optical methods. However, the performance of conventional protocols such as IMPROVE, STN, and EUSAAR differs for aerosol and geological samples due to different pyrolysis amounts and behaviors. Reflectance and transmittance pyrolysis corrections may be biased to various degrees depending on the sample matrix and temperature program, but in principle, it is most plausible that ECR > true EC > ECT. Reporting both ECR and ECT would help constrain the uncertainties in EC measurement if real EC can be measured by this method, and the difference between ECR and ECT provides a means for evaluating the performance (i.e., the accuracy) of a particular analysis protocol. In this study, a modified STN protocol with extended heating time in the inert atmosphere (STN120) shows smallest ECR–ECT range for soil and dust samples.

The IMPROVE protocol provides additional benefits of separating char and soot, and this facilitates the identification of EC sources. This should be a consideration for future research, including efforts to standardize analytical methods. Most of the EC concentrations derived from the thermal/optical methods in this study were much higher than those from CTO-375, a benchmark for measuring EC in geological material. CTO-375 EC is more relevant to the soot fraction of EC which does not evolve under a long-term heating in ambient air. 

## Supporting Information

Figure S1 **Sample locations for urban street dusts and surface soils in Baoji, China.**
(TIF)

Figure S2 **Comparison of total carbon (TC, unites of mg g^-1^) on filter samples measured with the IMPROVE-550, IMPROVE-675, STN60 and STN120 protocols performed in a DRI Model 2001 carbon analyzer.** Robust linear regression was used for all the analyses.
(TIF)

Figure S3 **Comparison of ECR and ECT (mg g^-1^) of urban street dusts (red dots) and surface soils (blue triangles) quantified by different protocols (see Supplement Figure S4 for separate regression analyses for dust and soil samples).**
(TIF)

Figure S4 **Detailed comparison of ECR and ECT (mg g^-1^) by different protocols for road dust (red dots, with high carbon loadings) and soil samples (blue triangle, with low carbon loadings) using robust linear regression analyses.**
(TIF)

Table S1 **Comparison of conventional IMPROVE (IMPROVE-550), STN (STN60) and EUSSAR protocols, as well as their modifications (IMPROVE-675 and STN120) tested in this study.**
(DOC)

Table S2 **Concentrations of carbon fractions (in mg g^-1^) measured with two IMPROVE (Interagency Monitoring of Protected Visual Environments) protocols, IMPROVE-550 and IMPROVE-675 (with the OC4 temperature of 550°C and 675°C, respectively) and two STN (Speciation Trends Network) protocols, STN60 and STN120 (with the time length for each OC step of 60 seconds and 120 seconds, respectively).**
(DOC)

Table S3 **Minimum reflectance (R) and transmittance (T) signals (units of mV) relative to the initial R and T for the four protocols.**
(DOC)

## References

[1] Masiello CA (2004) New directions in black carbon organic geochemistry. Marine Chemistry 92: 201-213. doi:10.1016/j.marchem.2004.06.043.

[2] Schmidt MWI, Noack AG (2000) Black carbon in soils and sediments: Analysis, distribution, implications, and current challenges. Global Biogeochemical Cycles 14: 777-793. doi:10.1029/1999GB001208.

[3] Goldberg ED (1985) Black carbon in the environment. New York: John Wiley & Sons, Inc.

[4] Han YM, Marlon J, Cao JJ, Jin ZD, An ZS (2012) Holocene linkages between char, soot, biomass burning and climate from Lake Daihai, China. Global Biogeochemical Cycles 26: GB4017 4010.1029/2012GB004413

[5] Ramanathan V, Carmichael G (2008) Global and regional climate changes due to black carbon. Nature Geoscience 1: 221-227. doi:10.1038/ngeo156.

[6] Janssen NAH, Gerlofs-Nijland ME, Lanki T, Salonen RO, Cassee F et al. (2012) Health Effects of Black Carbon. World Health Organization Report. WHO Regional Office for Europe, Copenhagen, Denmark.

[7] Lehmann J (2007) Bio-energy in the black. Frontiers in Ecology and the Environment 5: 381-387. Available online at: doi:10.1890/1540-9295(2007)5[381:BITB]2.0.CO;2

[8] Harvey OR, Kuo L-J, Zimmerman AR, Louchouarn P, Amonette JE et al. (2013) An Index-Based Approach to Assessing Recalcitrance and Soil Carbon Sequestration Potential of Engineered Black Carbons (Biochars). Environmental Science and Technology 46: 1415-1421. 10.1021/es2040398 22242866

[9] Han YM, Cao JJ, Yan BZ, Kenna TC, Jin ZD et al. (2011) Comparison of elemental carbon in lake sediments measured by TOR, TOT and CTO methods and 150-year pollution history in Eastern China. Environmental Science and Technology 45: 5287-5293. doi:10.1021/es103518c. 21591674

[10] Hammes K, Schmidt MWI, Smernik RJ, Currie LA, Ball WP et al. (2007) Comparison of quantification methods to measure fire-derived (black/elemental) carbon in soils and sediments using reference materials from soil, water, sediment and the atmosphere. Global Biogeochemical Cycles 21.

[11] Schmidt MWI, Skjemstad JO, Czimczik CI, Glaser B, Prentice KM et al. (2001) Comparative analysis of black carbon in soils. Global Biogeochemical Cycles 15: 163-167. doi:10.1029/2000GB001284.

[12] Watson JG, Chow JC, Chen L-WA (2005) Summary of organic and elemental carbon/black carbon analysis methods and intercomparisons. Aerosol and Air Quality Research 5: 65-102.

[13] Hitzenberger R, Petzold A, Bauer H, Ctyroky P, Pouresmaeil P et al. (2006) Intercomparison of thermal and optical measurement methods for elemental carbon and black carbon at an urban location. Environ Sci Technol 40: 6377-6383. doi:10.1021/es051228v. PubMed: 17120568. 17120568

[14] Subramanian R, Khlystov AY, Robinson AL (2006) Effect of peak inert-mode temperature on elemental carbon measured using thermal-optical analysis. Aerosol Science and Technology 40: 763-780. doi:10.1080/02786820600714403.

[15] Schauer JJ, Mader BT, Deminter JT, Heidemann G, Bae MS et al. (2003) ACE-Asia intercomparison of a thermal-optical method for the determination of particle-phase organic and elemental carbon. Environ Sci Technol 37: 993-1001. doi:10.1021/es020622f. PubMed: 12666931. 12666931

[16] Han YM, Han ZW, Cao JJ, Chow JC, Watson JG et al. (2008) Distribution and origin of carbonaceous aerosol over a rural high-mountain lake area, Northern China and its transport significance. Atmospheric Environment 42: 2405-2414. doi:10.1016/j.atmosenv.2007.12.020.

[17] Chow JC, Watson JG, Pritchett LC, Pierson WR, Frazier CA et al. (1993) The dri thermal/optical reflectance carbon analysis system: description, evaluation and applications in U.S. Air quality studies. Atmospheric Environment Part A General Topics 27: 1185-1201. doi:10.1016/0960-1686(93)90245-T.

[18] Han YN, Cao JJ, An ZS, Chow JC, Watson JG et al. (2007) Evaluation of the thermal/optical reflectance method for quantification of elemental carbon in sediments. Chemosphere 69: 526-533. doi:10.1016/j.chemosphere.2007.03.035. PubMed: 17498774. 17498774

[19] Birch ME (1998) Analysis of carbonaceous aerosols: interlaboratory comparison. Analyst 123: 851-857. doi:10.1039/a800028j. PubMed: 9709478. 9709478

[20] Peterson MR, Richards MH (2002) Thermal-optical-transmittance analysis for organic, elemental, carbonate, total carbon, and OCX2 in PM2.5 by the EPA/NIOSH method. In: Tropp RJ Pittsburgh, PA. Air and Waste Management Association.

[21] Cavalli F, Viana M, Yttri KE, Genberg J, Putaud JP (2010) Toward a standardised thermal-optical protocol for measuring atmospheric organic and elemental carbon: the EUSAAR protocol. Atmospheric. Measurement Techniques 3: 79-89.

[22] Chow JC, Watson JG, Chen LWA, Arnott WP, Moosmüller H (2004) Equivalence of elemental carbon by thermal/optical reflectance and transmittance with different temperature protocols. Environ Sci Technol 38: 4414-4422. doi:10.1021/es034936u. PubMed: 15382872. 15382872

[23] Han YM, Cao JJ, Posmentier ES, Chow JC, Watson JG et al. (2009) The effect of acidification on the determination of elemental carbon, char-, and soot-elemental carbon in soils and sediments. Chemosphere 75: 92-99. doi:10.1016/j.chemosphere.2008.11.044. PubMed: 19108866. 19108866

[24] Fung K (1990) Particulate Carbon Speciation by Mno2 Oxidation. Aerosol Science and Technology 12: 122-127. doi:10.1080/02786829008959332.

[25] Gustafsson O, Gschwend PM (1997) Soot as a strong partition medium for polycyclic aromatic hydrocarbons in aquatic systems. Molecular Markers in Environmental Geochemistry: 365-381.

[26] Nguyen TH, Brown RA, Ball WP (2004) An evaluation of thermal resistance as a measure of black carbon content in diesel soot, wood char, and sediment. Organic Geochemistry 35: 217-234. doi:10.1016/j.orggeochem.2003.09.005.

[27] Husain L, Khan AJ, Ahmed T, Swami K, Bari A et al. (2008) Trends in atmospheric elemental carbon concentrations from 1835 to 2005. Journal of Geophysical Research-Atmospheres 113. doi:10.1029/2007JD009398.

[28] Gustafsson O, Haghseta F, Chan C, MacFarlane J, Gschwend PM (1997) Quantification of the dilute sedimentary soot phase: Implications for PAH speciation and bioavailability. Environmental Science and Technology 31: 203-209. doi:10.1021/es960317s.

[29] Accardi-Dey A, Gschwend PM (2002) Assessing the combined roles of natural organic matter and black carbon as sorbents in sediments. Environmental Science and Technology 36: 21-29. doi:10.1021/es010953c. 11811485

[30] Chen L-WA, Chow JC, Watson JG, Schichtel BA (2012) Consistency of long-term elemental carbon trends from thermal and optical measurements in the IMPROVE network. Atmospheric. Measurement Techniques 5: 2329-2338.

[31] Han YM, Cao JJ, Chow JC, Watson JG, An ZS et al. (2007) Evaluation of the thermal/optical reflectance method for discrimination between char- and soot-EC. Chemosphere 69: 569-574. doi:10.1016/j.chemosphere.2007.03.024. PubMed: 17462705. 17462705

[32] Han YM, Cao JJ, Chow JC, Watson JG, An ZS et al. (2009) Elemental carbon in urban soils and road dusts in Xi'an, China and its implication for air pollution. Atmospheric Environment 43: 2464-2470. doi:10.1016/j.atmosenv.2009.01.040.

[33] Dutter R, Huber PJ (1981) Numerical methods for the nonlinear robust regression problem. Journal of Statistical Computation and Simulation 13: 79-113. doi:10.1080/00949658108810482.

[34] Chen LWA, Chow JC, Watson JG, Moosmuller H, Arnott WP (2004) Modeling reflectance and transmittance of quartz-fiber filter samples containing elemental carbon particles: Implications for thermal/optical analysis. Journal of Aerosol Science 35: 765-780. doi:10.1016/j.jaerosci.2003.12.005.

[35] Chow JC, Watson JG, Crow D, Lowenthal DH, Merrifield T (2001) Comparison of IMPROVE and NIOSH carbon measurements. Aerosol Science and Technology 34: 23-34. doi:10.1080/02786820119073.

[36] Novakov T, Corrigan CE (1995) Thermal Characterization of Biomass Smoke Particles. Mikrochimica Acta 119: 157-166. doi:10.1007/BF01244864.

[37] Cheng Y, Duan F-k, He K-b, Zheng M, Du Z-y et al. (2011) Intercomparison of Thermal-Optical Methods for the Determination of Organic and Elemental Carbon: Influences of Aerosol Composition and Implications. Environ Sci Technol 45: 10117-10123. PubMed: 22044188. 22044188 10.1021/es202649g

[38] Cheng Y, Duan F-k, He K-b, Du Z-y, Zheng M et al. (2012) Intercomparison of thermal-optical method with different temperature protocols: Implications from source samples and solvent extraction. Atmospheric Environment 61: 453-462. doi:10.1016/j.atmosenv.2012.07.066.

[39] Chow JC, Watson JG, Chen LWA, Chang MCO, Robinson NF et al. (2007) The IMPROVE-A temperature protocol for thermal/optical carbon analysis: maintaining consistency with a long-term database. Journal of the Air and Waste Management Association 57: 1014-1023. 17912920 10.3155/1047-3289.57.9.1014

[40] Gustafsson O, Bucheli TD, Kukulska Z, Andersson M, Largeau C et al. (2001) Evaluation of a protocol for the quantification of black carbon in sediments. Global Biogeochemical Cycles 15: 881-890. doi:10.1029/2000GB001380.

[41] Han YM, Cao JJ, Lee SC, Ho KF, An ZS (2010) Different characteristics of char and soot in the atmosphere and their ratio as an indicator for source identification in Xi'an, China. Atmospheric Chemistry and Physics 10: 595-607. doi:10.5194/acp-10-595-2010.

