# Supplementary figures and images for: Thermal/Optical Methods for Elemental Carbon Quantification in Soils and Urban Dusts: Equivalence of Different Analysis Protocols
Source: PLoS One. 2013 Dec 17;8(12):e83462. doi: 10.1371/journal.pone.0083462 (PMC3866270; doi:10.1371/journal.pone.0083462)

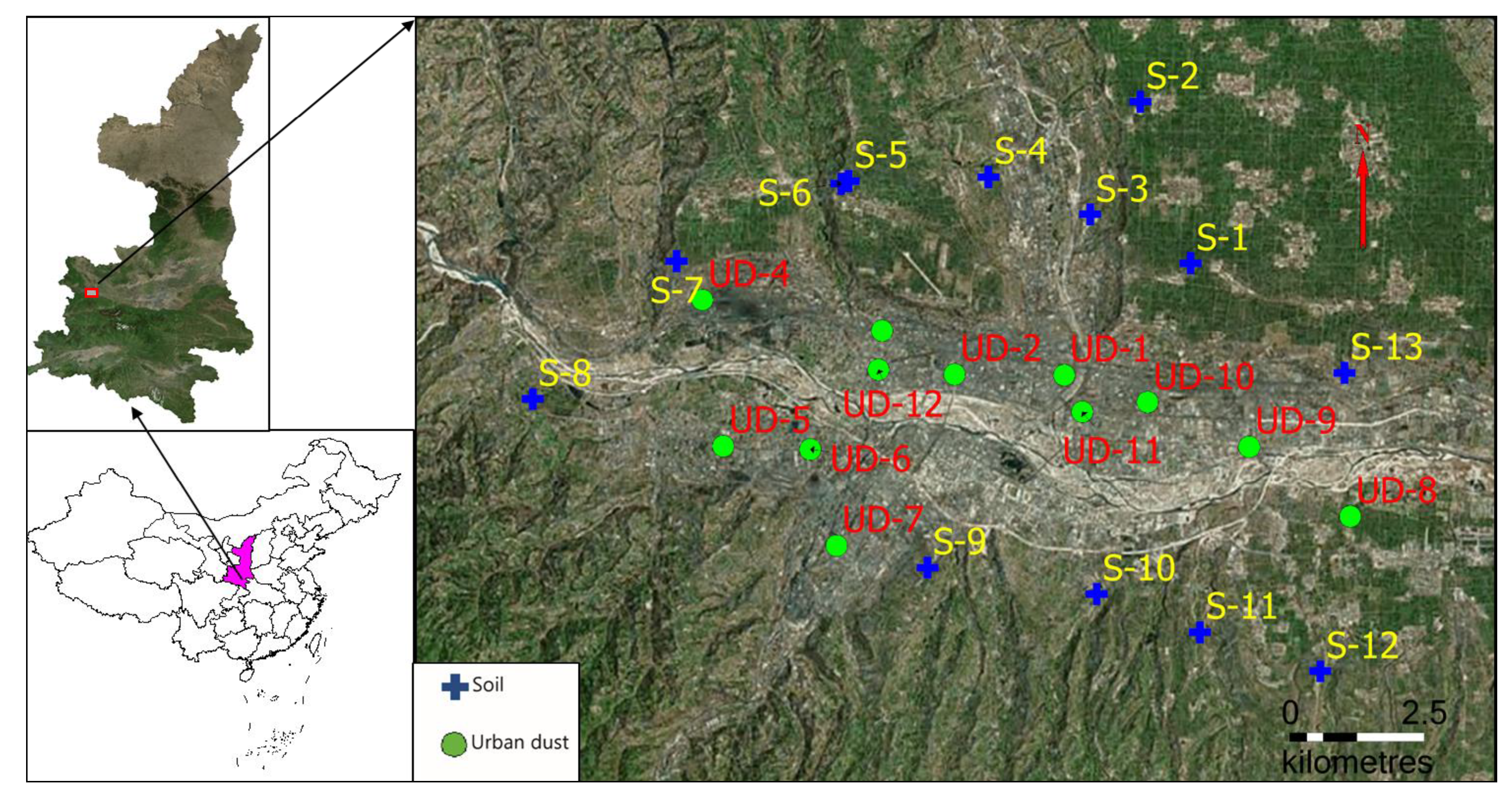

Supplement: Figure S1 — Sample locations for urban street dusts and surface soils in Baoji, China. (TIF) [file pone.0083462.s001.tif]

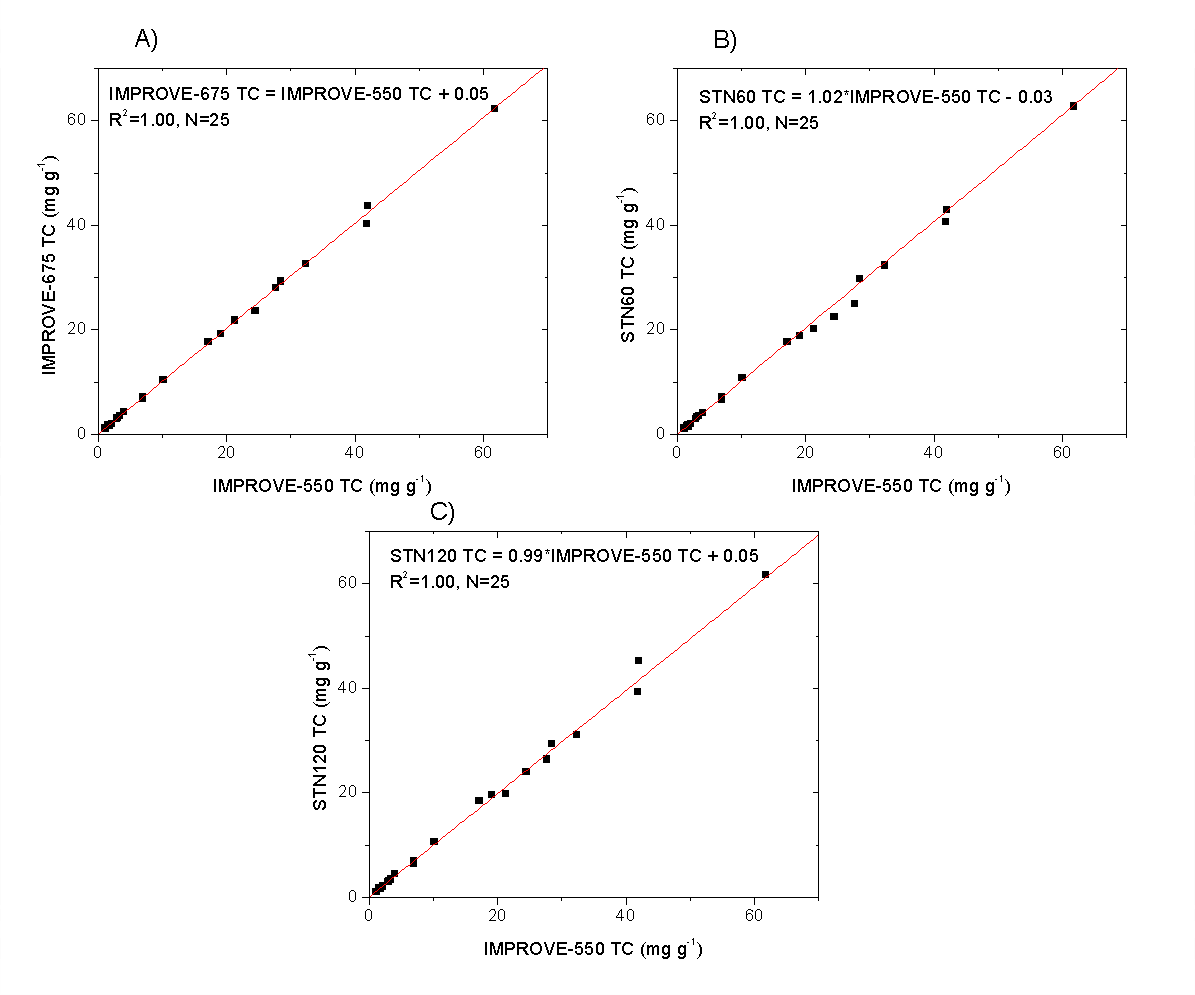

Supplement: Figure S2 — Comparison of total carbon (TC, unites of mg g-1) on filter samples measured with the IMPROVE-550, IMPROVE-675, STN60 and STN120 protocols performed in a DRI Model 2001 carbon analyzer. Robust linear regression was used for all the analyses. (TIF) [file pone.0083462.s002.tif]

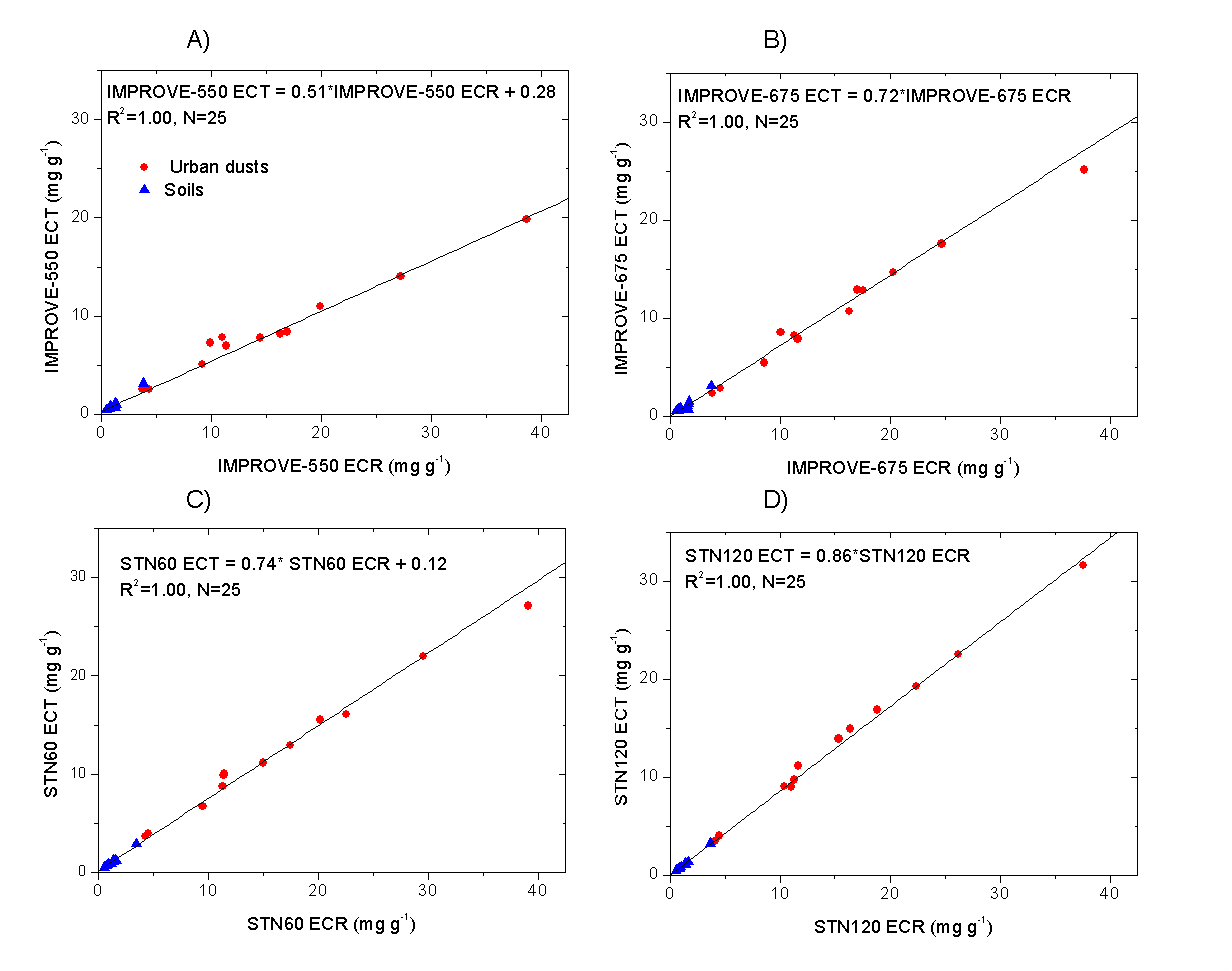

Supplement: Figure S3 — Comparison of ECR and ECT (mg g-1) of urban street dusts (red dots) and surface soils (blue triangles) quantified by different protocols (see Supplement Figure S4 for separate regression analyses for dust and soil samples). (TIF) [file pone.0083462.s003.tif]

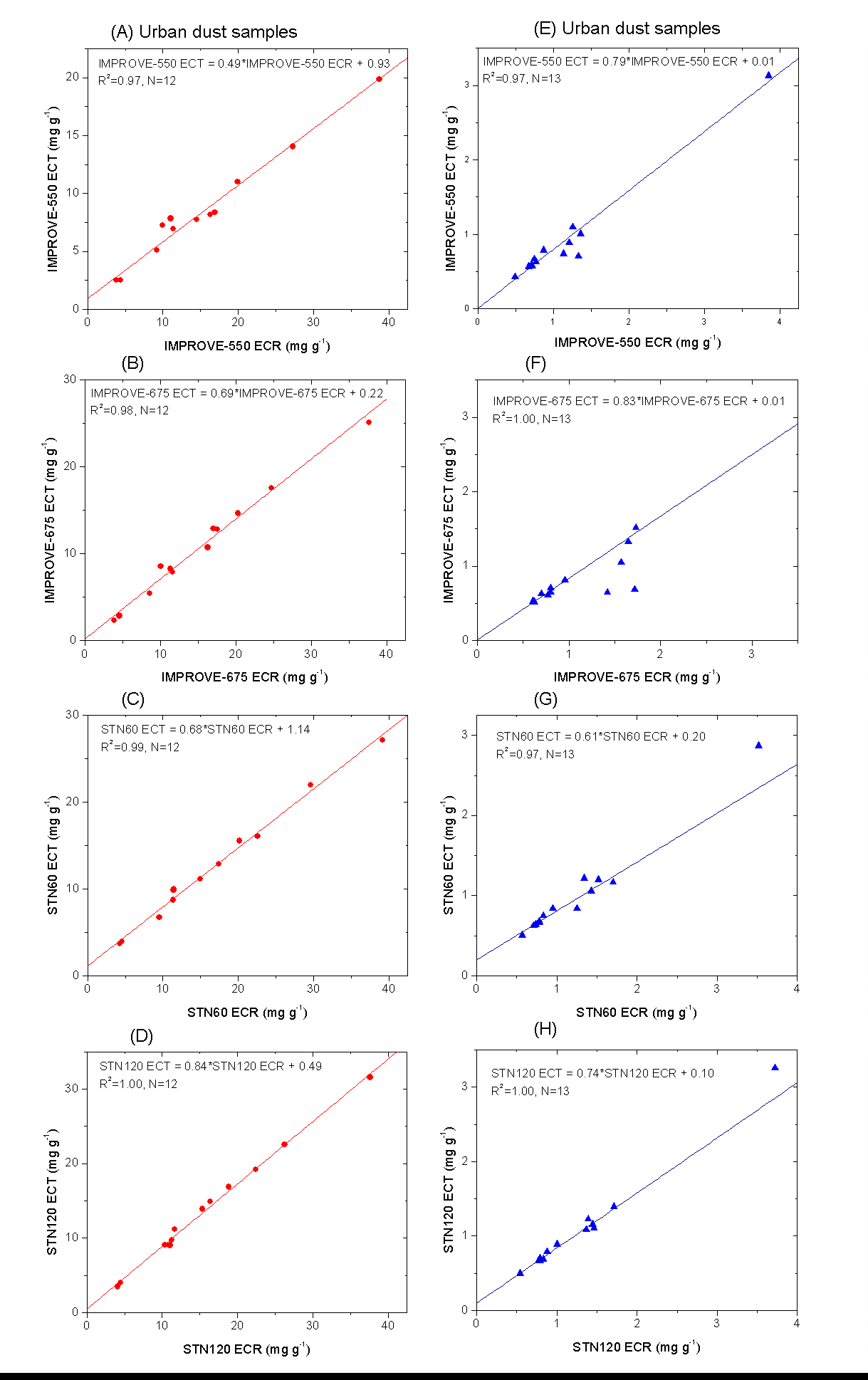

Supplement: Figure S4 — Detailed comparison of ECR and ECT (mg g-1) by different protocols for road dust (red dots, with high carbon loadings) and soil samples (blue triangle, with low carbon loadings) using robust linear regression analyses. (TIF) [file pone.0083462.s004.tif]
