# Supplementary material for: Thermal/Optical Methods for Elemental Carbon Quantification in Soils and Urban Dusts: Equivalence of Different Analysis Protocols
Source: PLoS One. 2013 Dec 17;8(12):e83462. doi: 10.1371/journal.pone.0083462 (PMC3866270; doi:10.1371/journal.pone.0083462)
Supplement: Table S1 — Comparison of conventional IMPROVE (IMPROVE-550), STN (STN60) and EUSSAR protocols, as well as their modifications (IMPROVE-675 and STN120) tested in this study. (DOC) [file pone.0083462.s005.doc]

**Table S1**. Comparison of conventional IMPROVE (IMPROVE-550), STN (STN60) and EUSSAR protocols, as well as their modifications (IMPROVE-675 and STN120) tested in this study.

|  | IMPROVE-550 | | | IMPROVE-675 | | | STN60 | | | STN120 | | | EUSSAR | | |
| --- | --- | --- | --- | --- | --- | --- | --- | --- | --- | --- | --- | --- | --- | --- | --- |
|  | Gas | Temp^1^ | Time^2^ | Gas | Temp | Time | Gas | Temp | Time | Gas | Temp | Time | Gas | Temp | Time |
| OC1 | He | 120 | 150-580 | He | 120 | 150-580 | He | 310 | 60 | He | 310 | 120 | He | 200 | 120 |
| OC2 | He | 250 | 150-580 | He | 250 | 150-580 | He | 480 | 60 | He | 480 | 120 | He | 300 | 150 |
| OC3 | He | 450 | 150-580 | He | 450 | 150-580 | He | 615 | 60 | He | 615 | 120 | He | 450 | 180 |
| OC4 | He | 550 | 150-580 | He | 675 | 150-580 | He | 900 | 90 | He | 900 | 120 | He | 650 | 180 |
|  | He | n/a | n/a | He | n/a | n/a | He | cool oven |  | He | cool oven |  | He | cool oven |  |
| EC1 | O_2_/He | 550 | 150-580 | O_2_/He | 675 | 150-580 | O_2_/He | 600 | 45 | O_2_/He | 600 | 90 | O_2_/He | 500 | 120 |
| EC2 | O_2_/He | 700 | 150-580 | O_2_/He | 700 | 150-580 | O_2_/He | 675 | 45 | O_2_/He | 675 | 90 | O_2_/He | 550 | 120 |
| EC3 | O_2_/He | 800 | 150-580 | O_2_/He | 800 | 150-580 | O_2_/He | 750 | 45 | O_2_/He | 750 | 90 | O_2_/He | 700 | 70 |
| EC4 | O_2_/He | n/a | n/a | O_2_/He | n/a | n/a | O_2_/He | 850 | 45 | O_2_/He | 850 | 90 | O_2_/He | 850 | 80 |
| EC5 | O_2_/He | n/a | n/a | O_2_/He | n/a | n/a | O_2_/He | 925 | 120 | O_2_/He | 925 | 120 | O_2_/He | n/a | n/a |
| Detection | Methanator / FID | | | | | | | | | | | | | | |
| Pyrolysis correction | Reflectance & Transmittance | | | | | | | | | | | | | | |

^1^Temperatures in degrees Celsius;

^2^Time in seconds.
