# Supplementary material for: Thermal/Optical Methods for Elemental Carbon Quantification in Soils and Urban Dusts: Equivalence of Different Analysis Protocols
Source: PLoS One. 2013 Dec 17;8(12):e83462. doi: 10.1371/journal.pone.0083462 (PMC3866270; doi:10.1371/journal.pone.0083462)
Supplement: Table S2 — Concentrations of carbon fractions (in mg g-1) measured with two IMPROVE (Interagency Monitoring of Protected Visual Environments) protocols, IMPROVE-550 and IMPROVE-675 (with the OC4 temperature of 550°C and 675°C, respectively) and two STN (Speciation Trends Network) protocols, STN60 and STN120 (with the time length for each OC step of 60 seconds and 120 seconds, respectively). (DOC) [file pone.0083462.s006.doc]

**Table S2**. Concentrations of carbon fractions (in mg g^-1^) measured with two IMPROVE (Interagency Monitoring of Protected Visual Environments) protocols, IMPROVE-550 and IMPROVE-675 (with the OC4 temperature of 550°C and 675°C, respectively) and two STN (Speciation Trends Network) protocols, STN60 and STN120 (with the time length for each OC step of 60 seconds and 120 seconds, respectively).

|  | IMPROVE(550) | | | |  | IMPROVE(675) | | |  | STN(60) | | |  | STN(120) | | |  | CTO-375 |
| --- | --- | --- | --- | --- | --- | --- | --- | --- | --- | --- | --- | --- | --- | --- | --- | --- | --- | --- |
| Sample ID | TC^1^ | ECR^2^ | ECT^3^ | soot |  | TC | ECR | ECT |  | TC | ECR | ECT |  | TC | ECR | ECT |  | EC |
| UD-1 | 41.79 | 27.26 | 14.05 | 3.46 |  | 40.26 | 24.73 | 17.57 |  | 40.64 | 29.59 | 21.98 |  | 39.32 | 26.24 | 22.53 |  | 1.10 |
| UD-2 | 24.50 | 9.19 | 5.10 | 2.12 |  | 23.72 | 8.57 | 5.45 |  | 22.55 | 9.52 | 6.71 |  | 24.03 | 10.39 | 9.08 |  | 1.74 |
| UD-3 | 27.64 | 14.49 | 7.77 | 2.43 |  | 28.02 | 16.28 | 10.71 |  | 24.95 | 15.00 | 11.15 |  | 26.41 | 15.35 | 13.90 |  | 0.74 |
| UD-4 | 21.35 | 11.40 | 6.94 | 3.20 |  | 21.84 | 11.62 | 7.89 |  | 20.13 | 11.36 | 8.74 |  | 19.76 | 11.03 | 9.02 |  | 1.48 |
| UD-5 | 19.14 | 11.05 | 7.85 | 4.36 |  | 19.28 | 11.32 | 8.25 |  | 18.93 | 11.43 | 9.88 |  | 19.68 | 11.28 | 9.74 |  | 0.80 |
| UD-6 | 17.17 | 9.96 | 7.25 | 2.11 |  | 17.70 | 10.06 | 8.53 |  | 17.70 | 11.48 | 10.00 |  | 18.52 | 11.66 | 11.17 |  | 1.10 |
| UD-7 | 42.02 | 19.94 | 11.01 | 2.62 |  | 43.65 | 20.32 | 14.65 |  | 42.93 | 22.57 | 16.06 |  | 45.20 | 22.41 | 19.23 |  | 0.95 |
| UD-8 | 7.03 | 3.81 | 2.51 | 0.64 |  | 7.16 | 3.86 | 2.33 |  | 7.18 | 4.29 | 3.67 |  | 6.99 | 4.07 | 3.49 |  | 1.53 |
| UD-9 | 10.17 | 4.38 | 2.54 | 0.60 |  | 10.46 | 4.56 | 2.87 |  | 10.78 | 4.56 | 3.96 |  | 10.59 | 4.48 | 4.02 |  | 0.35 |
| UD-10 | 61.74 | 38.72 | 19.84 | 7.95 |  | 62.25 | 37.68 | 25.12 |  | 62.74 | 39.12 | 27.12 |  | 61.65 | 37.59 | 31.62 |  | 1.86 |
| UD-11 | 32.36 | 16.92 | 8.38 | 1.52 |  | 32.59 | 17.04 | 12.90 |  | 32.35 | 17.44 | 12.89 |  | 31.13 | 16.41 | 14.93 |  | 3.14 |
| UD-12 | 28.52 | 16.28 | 8.18 | 1.77 |  | 29.30 | 17.57 | 12.83 |  | 29.76 | 20.18 | 15.53 |  | 29.33 | 18.83 | 16.87 |  | 0.63 |
| S-1 | 1.73 | 0.67 | 0.57 | 0.11 |  | 1.75 | 0.62 | 0.52 |  | 1.80 | 0.78 | 0.67 |  | 1.82 | 0.79 | 0.67 |  | 0.13 |
| S-2 | 2.98 | 1.26 | 1.10 | 0.11 |  | 3.07 | 1.73 | 1.52 |  | 2.95 | 1.34 | 1.22 |  | 3.00 | 1.39 | 1.23 |  | 0.10 |
| S-3 | 3.44 | 1.36 | 1.01 | 0.13 |  | 3.59 | 1.57 | 1.05 |  | 3.56 | 1.52 | 1.20 |  | 3.32 | 1.45 | 1.16 |  | 0.09 |
| S-4 | 3.09 | 1.21 | 0.89 | 0.18 |  | 3.12 | 1.65 | 1.33 |  | 3.32 | 1.43 | 1.06 |  | 3.05 | 1.37 | 1.09 |  | 0.13 |
| S-5 | 1.65 | 0.71 | 0.59 | 0.08 |  | 1.66 | 0.77 | 0.61 |  | 1.67 | 0.78 | 0.68 |  | 1.60 | 0.79 | 0.70 |  | 0.08 |
| S-6 | 1.77 | 0.77 | 0.63 | 0.11 |  | 1.68 | 0.80 | 0.65 |  | 1.51 | 0.71 | 0.63 |  | 1.66 | 0.77 | 0.67 |  | 0.15 |
| S-7 | 2.17 | 0.87 | 0.79 | 0.09 |  | 2.07 | 0.96 | 0.81 |  | 1.96 | 0.95 | 0.84 |  | 2.09 | 1.00 | 0.89 |  | 0.11 |
| S-8 | 1.62 | 0.75 | 0.67 | 0.07 |  | 1.67 | 0.80 | 0.71 |  | 1.65 | 0.83 | 0.75 |  | 1.74 | 0.88 | 0.79 |  | 0.10 |
| S-9 | 6.93 | 3.85 | 3.13 | 0.65 |  | 6.75 | 3.74 | 3.12 |  | 6.56 | 3.52 | 2.87 |  | 6.34 | 3.72 | 3.26 |  | 0.80 |
| S-10 | 1.61 | 0.72 | 0.58 | 0.11 |  | 1.73 | 0.70 | 0.63 |  | 1.53 | 0.74 | 0.64 |  | 1.72 | 0.83 | 0.69 |  | 0.11 |
| S-11 | 3.35 | 1.13 | 0.74 | 0.29 |  | 3.46 | 1.42 | 0.65 |  | 3.46 | 1.25 | 0.84 |  | 3.53 | 1.46 | 1.11 |  | 0.20 |
| S-12 | 3.99 | 1.33 | 0.71 | 0.26 |  | 4.30 | 1.72 | 0.69 |  | 4.12 | 1.70 | 1.17 |  | 4.49 | 1.71 | 1.40 |  | 0.12 |
| S-13 | 1.16 | 0.49 | 0.43 | 0.08 |  | 1.13 | 0.61 | 0.53 |  | 1.16 | 0.57 | 0.51 |  | 1.11 | 0.54 | 0.50 |  | 0.13 |

^1^TC is the total carbon on the filter after acid pretreatment;

^2^ECR is elemental carbon with reflectance optical correction;

^3^ECT is elemental carbon with transmittance optical correction.
