# Supplementary material for: Thermal/Optical Methods for Elemental Carbon Quantification in Soils and Urban Dusts: Equivalence of Different Analysis Protocols
Source: PLoS One. 2013 Dec 17;8(12):e83462. doi: 10.1371/journal.pone.0083462 (PMC3866270; doi:10.1371/journal.pone.0083462)
Supplement: Table S3 — Minimum reflectance (R) and transmittance (T) signals (units of mV) relative to the initial R and T for the four protocols. (DOC) [file pone.0083462.s007.doc]

**Table S3**. Minimum reflectance (R) and transmittance (T) signals (units of mV) relative to the initial R and T for the four protocols.

|  | Improve-550 | | Improve-675 | | STN60 | | STN120 | |
| --- | --- | --- | --- | --- | --- | --- | --- | --- |
| Sample ID | ∆R_min_ | ∆T_min_ | ∆R_min_ | ∆T_min_ | ∆R_min_ | ∆T_min_ | ∆R_min_ | ∆T_min_ |
| BJ UD-1 | -118 | -351 | -210 | -417 | -152 | -397 | -172 | -411 |
| BJ UD-2 | -174 | -506 | -213 | -508 | -183 | -530 | -203 | -555 |
| BJ UD-3 | -176 | -475 | -232 | -486 | -239 | -541 | -215 | -515 |
| BJ UD-4 | -147 | -389 | -227 | -438 | -166 | -474 | -167 | -478 |
| BJ UD-5 | -158 | -402 | -235 | -445 | -172 | -482 | -176 | -491 |
| BJ UD-6 | -171 | -366 | -245 | -402 | -182 | -413 | -187 | -414 |
| BJ UD-7 | -169 | -476 | -216 | -480 | -215 | -506 | -236 | -523 |
| BJ UD-8 | -87 | -239 | -127 | -283 | -80 | -233 | -83 | -222 |
| BJ UD-9 | -98 | -256 | -142 | -301 | -91 | -248 | -95 | -233 |
| BJ UD-10 | -134 | -382 | -242 | -453 | -185 | -431 | -201 | -446 |
| BJ UD-11 | -125 | -410 | -184 | -411 | -161 | -410 | -184 | -427 |
| BJ UD-12 | -145 | -376 | -241 | -437 | -203 | -477 | -196 | -450 |
| BJ S-1 | -130 | -178 | -135 | -198 | -137 | -179 | -143 | -163 |
| BJ S-2 | -116 | -110 | -119 | -113 | -113 | -97 | -106 | -105 |
| BJ S-3 | -137 | -192 | -141 | -203 | -136 | -186 | -128 | -195 |
| BJ S-4 | -142 | -202 | -171 | -229 | -131 | -206 | -126 | -202 |
| BJ S-5 | -114 | -132 | -120 | -157 | -117 | -140 | -118 | -127 |
| BJ S-6 | -116 | -152 | -118 | -166 | -129 | -153 | -126 | -155 |
| BJ S-7 | -113 | -106 | -120 | -132 | -113 | -106 | -101 | -104 |
| BJ S-8 | -115 | -149 | -127 | -163 | -115 | -156 | -102 | -146 |
| BJ S-9 | -112 | -153 | -123 | -139 | -114 | -189 | -113 | -177 |
| BJ S-10 | -128 | -178 | -127 | -170 | -102 | -181 | -115 | -174 |
| BJ S-11 | -115 | -155 | -125 | -142 | -117 | -193 | -116 | -180 |
| BJ S-12 | -159 | -231 | -184 | -255 | -167 | -255 | -154 | -264 |
| BJ S-13 | -115 | -158 | -130 | -158 | -114 | -174 | -115 | -167 |
